# Altered sirtuin expression is associated with node-positive breast cancer

**DOI:** 10.1038/sj.bjc.6603384

**Published:** 2006-09-26

**Authors:** N Ashraf, S Zino, A MacIntyre, D Kingsmore, A P Payne, W D George, P G Shiels

**Affiliations:** 1 Division of Cancer Sciences and Molecular Pathology, Department of Surgery, University of Glasgow, Western Infirmary Glasgow, 44 Church Street, Glasgow G11 6NT, UK; 2 Renal Transplant Unit, Western Infirmary Glasgow, Glasgow, UK; 3 IBLS, University of Glasgow, Glasgow, UK

**Keywords:** breast cancer, sirtuins, gene expression

## Abstract

Sirtuins are genes implicated in cellular and organismal ageing. Consequently, they are speculated to be involved in diseases of ageing including cancer. Various cancers with widely differing prognosis have been shown to have differing and characteristic expression of these genes; however, the relationship between sirtuin expression and cancer progression is unclear. In order to correlate cancer progression and sirtuin expression, we have assessed sirtuin expression as a function of primary cell ageing and compared sirtuin expression in normal, ‘nonmalignant’ breast biopsies to breast cancer biopsies using real-time polymerase chain reaction (PCR). Levels of SIRT7 expression were significantly increased in breast cancer (*P*<0.0001). Increased levels of SIRT3 and SIRT7 transcription were also associated with node-positive breast cancer (*P*<0.05 and *P*<0.0001, respectively). This study has demonstrated differential sirtuin expression between nonmalignant and malignant breast tissue, with consequent diagnostic and therapeutic implications.

Breast cancer is the most common cancer to affect women, with over 40 000 new cases being detected and around 13 000 women dying from the disease in Britain each year (CancerStats, 2005). The prognosis of breast cancer has traditionally been based on prognostic factors, such as tumour size, nodal status and histological grade, that have been derived from population-based studies. To improve the accuracy of predicting prognosis, scoring systems that combine these factors have been developed, such as the Nottingham Prognostic Index (NPI) (Galea *et al*, 1992). However, these prognostic factors are less accurate predictors of prognosis in an individual patient, owing to widely differing disease behaviour that ranges from slow-growing, nonmetastasising disease with an excellent prognosis, to fast-growing disease with a propensity for early metastasis and rapid death.

In contrast to the well-defined behaviour of breast cancer in a population, the molecular aetiology of cancer initiation and progression is not fully understood. Genes involved in biological ageing may be informative in this context, as the incidence of cancer increases as a function of chronological age. Specifically, genes involved in NAD^+^-dependent protein deacetylation may have a role to play in cancer pathogenesis (Marks *et al*, 2001; Johnstone, 2002; Neumeister *et al*, 2002). These genes comprise the sirtuins, orthologues of the yeast silent information regulator 2 (*SIR2*) family of genes. Sirtuins are a highly conserved set of genes found in organisms ranging from bacteria to man (Brachmann *et al*, 1995; Frye, 2000), involved in a variety of essential cell processes, including ageing, preventing differentiation, apoptosis and resistance to metabolic stress (Vaziri *et al*, 2001; Dryden *et al*, 2003). We have previously hypothesised that sirtuins, which link the functions of the mitochondrion, telomere nucleo-protein complex and ribosome production (the MTR), may be important contributors to a wide range of ageing related diseases including cancer (Shiels and Davies, 2003). Consequently, we have undertaken a study designed to investigate sirtuin expression in breast cancer. Seven sirtuins (*SIRT1–7*) have so far been reported in humans. Of these, *SIRT2* and *7* have been associated with particular cancers with widely varying natural histories, *SIRT2* with gliomas and *SIRT7* with thyroid cancer (de Nigris *et al*, 2002; Frye, 2002; Hiratsuka *et al*, 2003). Given the association of sirtuin expression with clinical behaviour, we investigated sirtuin expression in breast cancer to determine if changes in the transcriptional expression of individual sirtuins correlated with malignancy and disease stage.

## MATERIALS AND METHODS

### Cell culture

Sirtuin expression was initially studied in replicatively ageing mammary epithelial cell lines. These included a primary mammary epithelial cell line (HMEC) obtained from Clonetics, UK, an immortalized nontumorigenic MCF-12A breast cell line from the Department of Pathology, University of Glasgow and a tumorigenic MCF-7 breast cancer cell line from the Department of Radiation Oncology, University of Glasgow. The HMECs were grown in mammary epithelial cell growth medium (MGEM) supplemented with bovine pituitary extract (BPE), epidermal growth factor, insulin, GA-1000 and hydrocortisone. The MCF-12A cells were grown in a 1 : 1 mixture of Dulbecco's modified Eagle's medium and Ham's F12 medium, supplemented with 5% foetal calf serum, 20 ng ml^−1^ epidermal growth factor, 100 ng ml^−1^ cholera toxin, 0.01 mg ml^−1^ bovine insulin and 500 ng ml^−1^ hydrocortisone. The MCF-7 cells were grown in RPMI medium with 10% foetal calf serum.

### Biopsies

Archival breast biopsies stored in liquid nitrogen and held by the Department of Surgery, Western Infirmary, Glasgow, were used for this study. These biopsies came from patients with potentially curable, invasive ductal adenocarcinomas who had given permission for the storage and future analysis of samples of tissue. All studies were undertaken with the approval of the local NHS ethics committee.

### RNA extraction, DNase treatment and cDNA synthesis

RNA extraction was performed using TRIzol® (Life Technologies, UK) on both the mammary epithelial cells and biopsies. Breast glandular tissue was carefully dissected free from surrounding fatty tissue. TRIzol® (1 ml) was added per 50 mg of tissue. Tissue was then homogenised using a mechanical tissue homogenizer at 0°C. RNA was DNase treated with a commercially available kit (Ambion, UK) according to the manufacturer's recommendations. Then, 2 *μ*g of RNA was reverse transcribed into cDNA using the Superscript™ First Strand Synthesis System (Invitrogen).

### Taqman® polymerase chain reaction (PCR)

Taqman® PCR was carried out on duplicate cDNA samples using an ABI Prism 7700 sequence detector in a 25 *μ*l reaction. The mixture consisted of 20 ng of cDNA, 300 nM of each forward and reverse primer, 200 nM Taqman® probe, 12.5 *μ*l of Taqman® Universal PCR Master Mix (Eurogentech, UK) and H_2_O. Cycle conditions were 50°C for 2 min, 95°C for 10 min followed by 40 cycles of 95°C for 15 s and 60°C for 1 min. Sequences of forward and reverse primers and probes used are shown below (Table 1).

### Senescence-associated *β*-galactosidase assay

The senescence-associated-*β*-galactosidase (SA-*β*-Gal) assay detects the presence of SA-*β*-Gal, specifically at pH 6, as an established histochemical marker of cellular senescence (Dimri *et al*, 1995). An assessment of the level of SA-*β*-Gal expression in all cultures was therefore determined at pH 6 following the method of Dimri *et al* (1995), with a pH 4 positive control included in all instances.

### Statistical analysis

The analysis of variance (ANOVA) was used to determine whether changes in sirtuin expression with replicative ageing were significant. The comparative ΔΔ*C*_T_ method was employed to quantify relative gene expression for the Taqman® studies. Analysis of individual gene expression between each of biopsy groups (i.e., normal, and node-positive and negative-breast biopsies) was performed using ANOVA. *Post hoc* comparisons were performed using the independent Student's *t*-test. Differences in gene expression between the biopsy groups were calculated along with the 95% confidence interval (CI). Results are presented in figures displaying mean relative gene expression and the standard error of the mean.

## RESULTS

**Figure 1:**
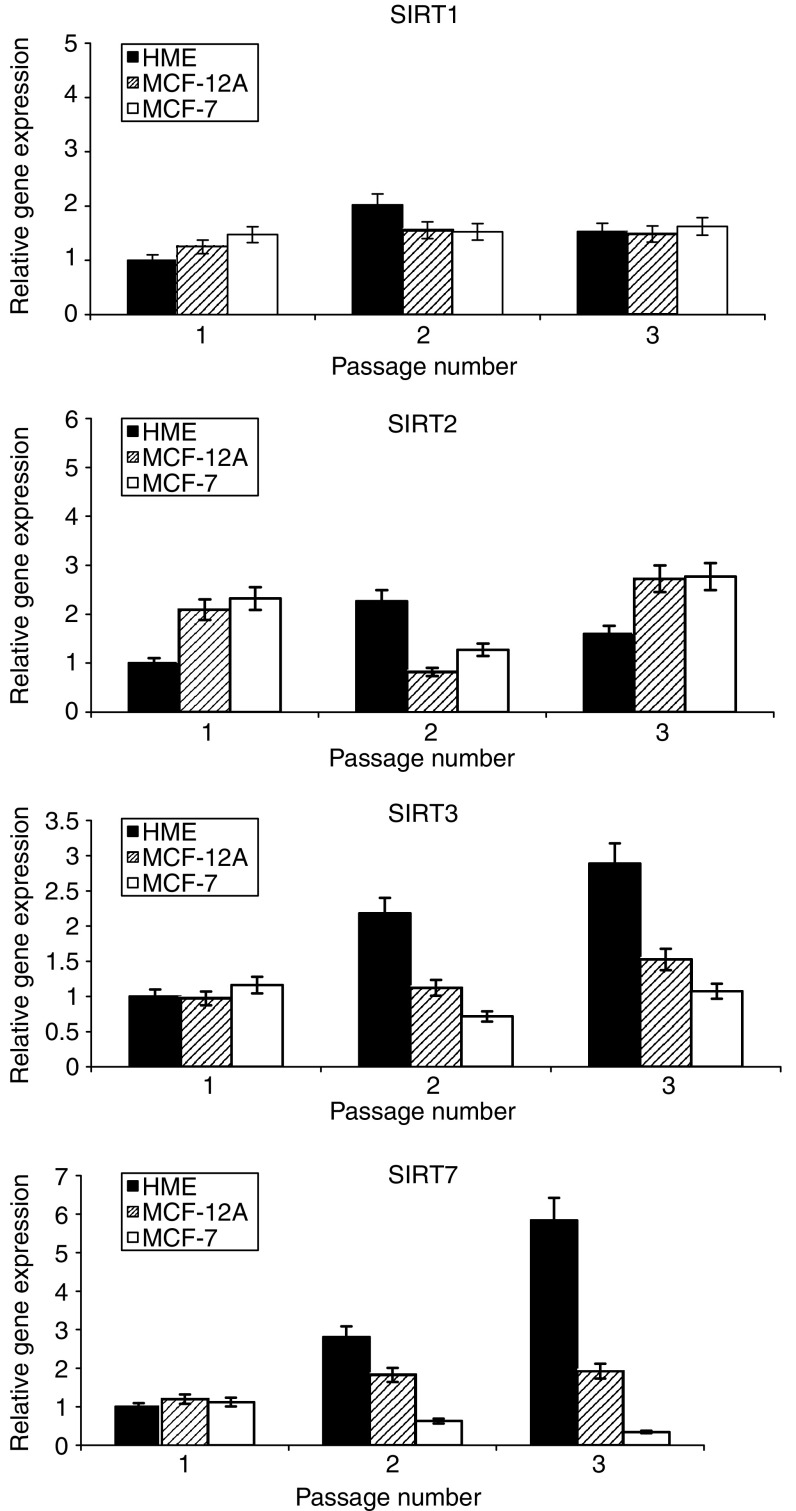
Sirtuin expression in serially passaged mammary epithelial cell cultures. The figure shows relative sirtuin gene expression in serially passaged cells relative to *18 s rRNA* gene expression. Mean data are plotted ±s.d.

Sirtuin gene expression in primary and immortalised cells was observed for change over time in culture. Transcriptional expression for Sirtuins 1, 2, 3 and 7 was undertaken in serially passaged primary breast cells (HMECs) and in immortalised nontumorigenic (MCF-12A) and tumorigenic (MCF-7) mammary epithelial cell lines. This was performed with a view to determining the transcriptional profile of sirtuins as a function of time spent in culture and hence biological ageing. *SIRT3* and *SIRT7* demonstrated an increase in transcriptional expression with increasing passage in HMECs (Figure 1). This observation was not repeated for *SIRT1* and *SIRT2*, nor did any sirtuin analysed showed a change in transcriptional expression in either of the immortalised cell lines (Figure 1).

**Figure 2:**
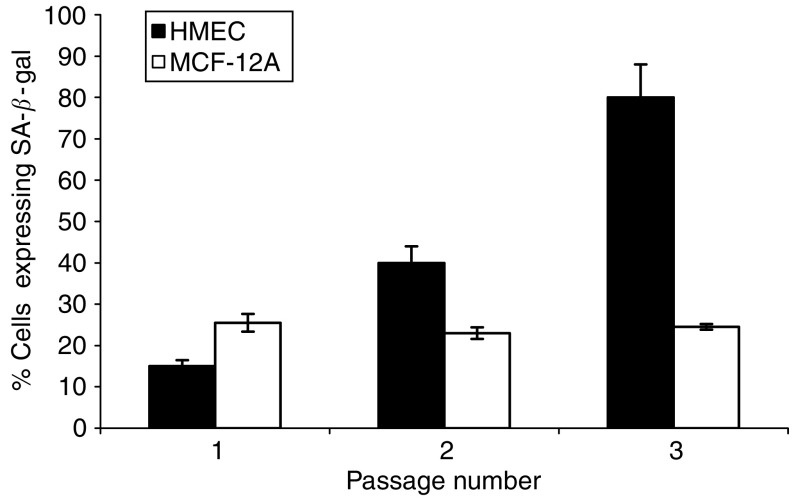
Senescence associated-*β-*galactosidase expression in serially passaged primary and immortalised mammary epithelial cells. Mean SA-*β*-Gal expression is shown ±s.d.

**Figure 3:**
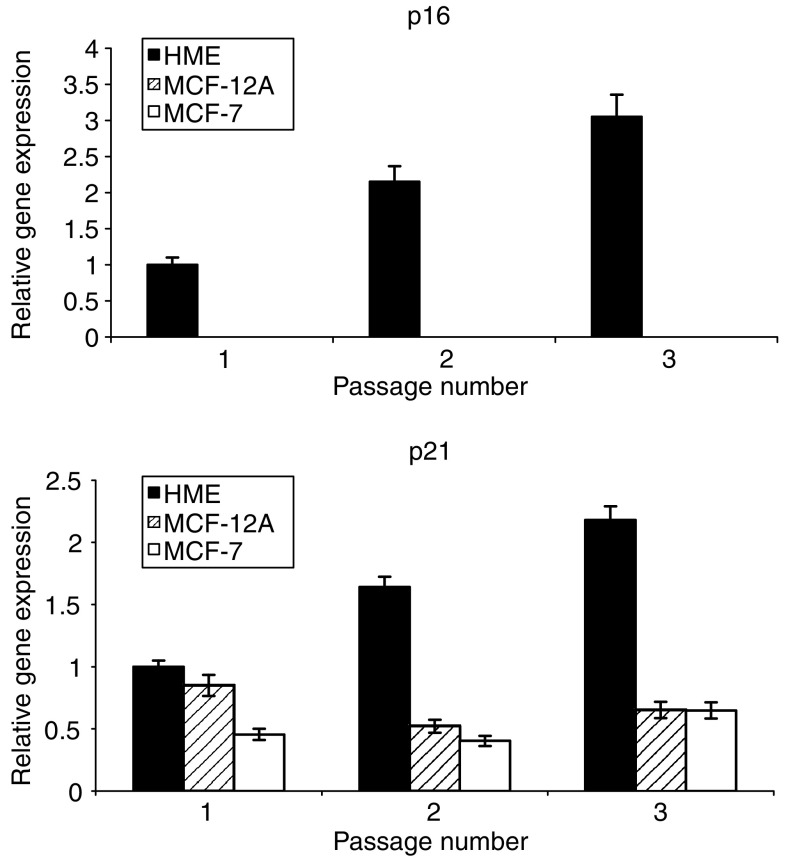
*p16* and *p21* expression in successively passaged breast cells. Histograms show the relative gene expression for *p16* and *p21* relative to *18 s rRNA* expression in primary cell cultures and immortalized breast cell lines. Data is plotted as means±s.em.

The increase in *SIRT3* and *SIRT7* expression was concurrent with an increase in SA-*β*-Gal expression in the culture with increasing passage, indicating the presence of increasing amounts of senescent cells in the population (Figure 2). Neither of the immortalised cell lines displayed a similar increase in SA-*β*-Gal expression. The acquisition of the senescent phenotype by the HMECs in association with serial passage was confirmed by a transcriptional analysis of the cellular stress and senescence genes *p16*^INK4a^ and *p21*^CIP1^ (Figure 3). The expression of *p16*^INK4a^ increased with serial passage only in the primary cell line, as did expression for *p21*^CIP1^, indicating that the transcriptional expression data for the sirtuins was associated with the generation of a senescent state in these cells.

### Biopsies

The pathological characteristics of the breast biopsies used in this study were selected to include equal distribution by nodal status (12 lymph node positive and 12 lymph node negative), and tumour grade (Table 2). A total of 24 breast cancer samples were chosen and matched with 21 ‘normal’, nonmalignant breast tissue biopsies from the breasts of cancer patients. These were then assessed for sirtuin expression levels.

**Figure 4:**
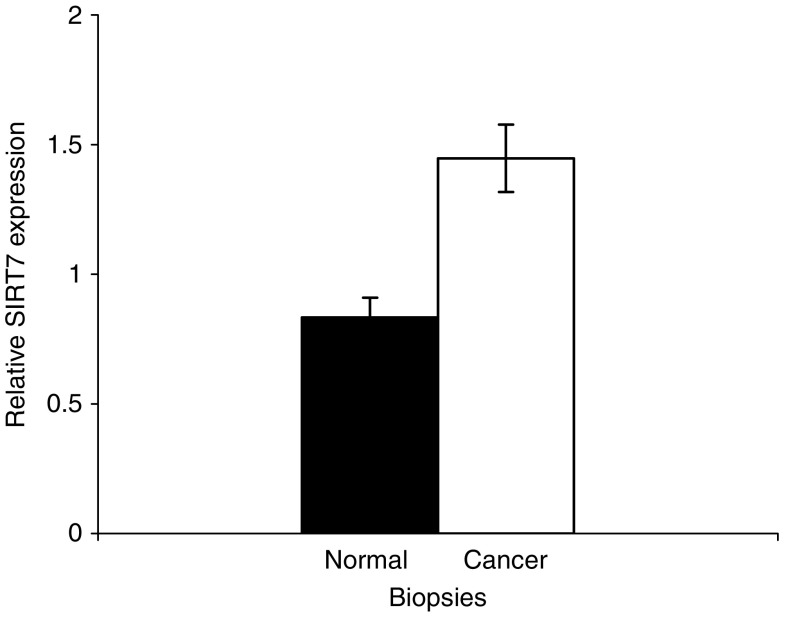
*SIRT7* expression in normal and breast cancer biopsies. HIstograms show relative sirtuin gene expression in nonmalignant and malignant breast biopsies, relative to an *18 s rRNA* control. Data show mean sirtuin expression ±s.e.m.

**Figure 5:**
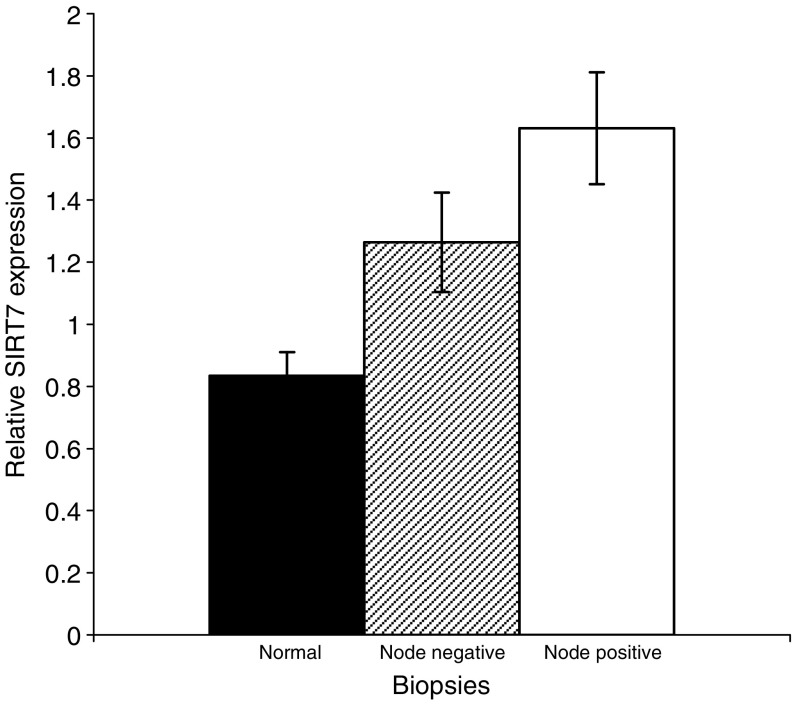
*SIRT7* expression in breast biopsies by nodal status. The histogram shows relative sirtuin gene expression in nonmalignant and lymph node-positive and -negative breast cancer biopsies. Data are plotted as mean sirtuin expression ±s.e.m.

Relative *SIRT7* gene expression was significantly greater in biopsies from breast cancers compared to ‘normal’, nonmalignant breast tissue (1.74 (95% CI 1.37–2.10), *P*<0.001, Figure 4). The relative expression of *SIRT7* was also significantly higher in node-positive compared to node-negative cancers (1.96, (95% CI 1.54–2.38) *vs* 1.52 (95% CI 1.13–1.90), *P*<0.001, Figure 5).

**Figure 6:**
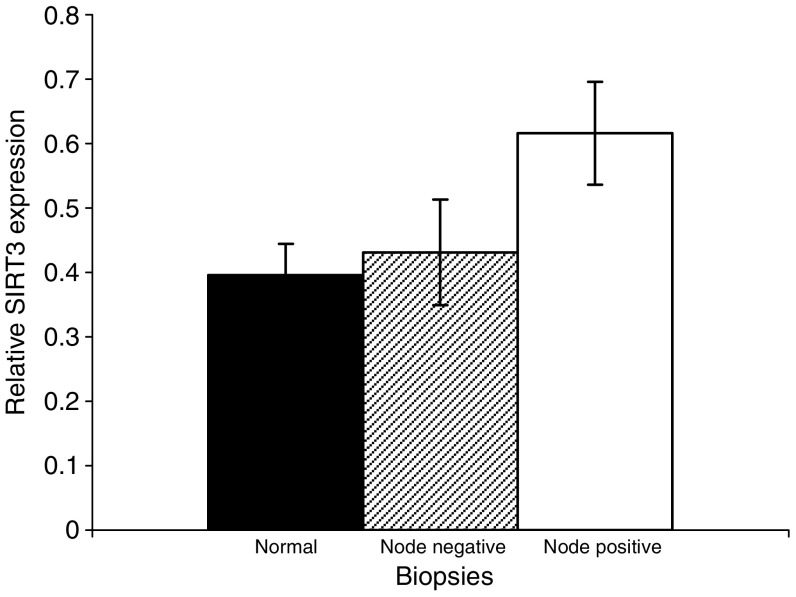
*SIRT3* expression in breast biopsies by nodal status. The histogram shows relative sirtuin gene expression in nonmalignant and lymph node-positive and -negative breast cancer biopsies. Data are plotted as mean sirtuin expression ±s.e.m.

No significant differences in *SIRT1*, *SIRT2* and *SIRT3* gene expression were observed in a simple comparison of biopsies from breast cancers with ‘normal’ breast tissue (data not shown). However, analogous to *SIRT7* expression, *SIRT3* expression was also significantly higher in lymph node-positive breast cancer biopsies when compared to ‘normal’ breast biopsies (1.56, 95% C.I. 1.10–2.01, *P*<0.05) (Figure 6).

Sirtuin expression was similarly compared by grade, oestrogen receptor status and the presence of lymphovascular invasion in both node-positive and node-negative cancers. No significant differences in sirtuin expression were seen using these factors (data not shown).

## DISCUSSION

We undertook this study to assess the possible association of changes in the transcriptional expression of individual sirtuin genes with breast cancer. Firstly, we investigated sirtuin gene expression in both primary and immortalised cells with a view to determining how cellular ageing and immortalisation influenced their transcription profile. Consequently, we observed *SIRT3* and *SIRT7* transcription to increase in primary cells as they approach senescence (Figure 1), concurrent with an increase in *p16*^*INK4a*^ and *p21*^*CIP1*^ expression (Figure 3) and the accumulation of SA-*β*-Gal-containing cells (Figure 2). Secondly, we demonstrated a significant *in vivo* association between altered *SIRT3* and *SIRT7* transcription and malignant breast disease (Figures 4, 5 and 6).

A link between the transcriptional expression of genes involved in biological ageing and cancer is pertinent. Individual sirtuins are respondents to intracellular redox changes and cell stress and hence biological ageing. In yeast, *SIR2* provides a functional link between the telomere and ribosomal DNA cluster integrity, thus impacting on ribosome production and enabling the cell to respond to stress induced redox state changes in the mitochondrion (Guarente, 2000). Consequently, damage responses in the cell can be modulated to match the degree of insult. In man, individual sirtuins may provide separate functional components of this process (Shiels and Davies, 2003). This is intriguing, in the light of the observations that *SIRT7* localises to the nucleolus and *SIRT3* localises to the mitochondrion (Onyango *et al*, 2002; Michishita *et al*, 2005). These subcellular locations, along with the telomere nucleoprotein complex, provide the components of a functional trinity, we have termed the MTR, that senses, assesses and signals damage (Shiels and Davies, 2003; Shiels and Jardine, 2003). Our observations that *SIRT3* and *SIRT7* expression is increased as primary mammary epithelial cells approach senescence and are also increased in node-positive breast cancer is in keeping with the MTR hypothesis.

As *SIRT7* expression is invariably elevated in thyroid cancer cell lines and biopsies, increased expression of *SIRT7* may represent an important step in malignant transformation (de Nigris *et al*, 2002; Frye, 2002). Indeed, *SIRT7* has recently been shown to activate Pol I encouraging growth and proliferation (Ford *et al*, 2006). In this study, we have shown that increased *SIRT7* expression is also observed in breast cancer, and may therefore be postulated, in a similar manner, to be required for malignant transformation (Figure 4). Furthermore, *SIRT7* expression is also associated with nodal invasion and therefore locally aggressive disease (Figure 5). Whether this increased expression contributes to the increased proliferative potential of mammary cancer cells or reflects a molecular change necessary for breast cancer tumorigenesis is undetermined. Alternatively, it may reflect a compensatory mechanism as a consequence of the increased proliferative state. This is supported by observations on the activity of the yeast sirtuin orthologue, *SIR2*. Nucleolar *SIR2* activity is a limiting factor for determining when the cell undergoes senescence (Guarente, 1999). The high proliferative rate in many tumours often results in them having shortened telomeres as a consequence of rapid cell turnover outstripping telomerase-mediated telomere repair. This may, in analogy with the situation in yeast (Guarente, 1999), lead to an upregulation of sirtuin activity in an attempt to stabilise the nucleolus. The increased *SIRT3* and *SIRT7* transcription in primary mammary epithelial cells, as a consequence of increased oxidant load with *in vitro* growth, is supportive of such a scenario. The association of elevated *SIRT7* expression in node-positive tumours, which have a greater recurrence and poorer survival, suggests that this gene may prove to be a good marker of disease progression and tumour behaviour.

There was no overall difference observed in *SIRT3* expression between breast cancer and normal breast tissue. However, on subdividing the cancer biopsies by nodal status, *SIRT3* expression, like that of *SIRT7*, was greater in node-positive breast cancer compared to normal breast tissue (Figure 6). *SIRT3* has been shown to specifically targeted and converted into its active form within the mitochondria (Dryden *et al*, 2003; North *et al*, 2003). Cumulative mitochondrial damage contributes to a fall in relative nicotinamide adenine dinucleotide (NAD) levels, and concomitant fall in *SIRT3* activity, and is associated with growth arrest, senescence and apoptosis (Shiels and Davies, 2003). Analogous to *SIRT1* and *SIRT2*, *SIRT3* may also function to provide a growth and survival advantage. Indeed, variability of the *SIRT3* gene has been linked to survival in the elderly (Rose *et al*, 2003). The increased expression of *SIRT3* seen in lymph node-positive tumours may, therefore, contribute to survival of these more aggressive tumours.

Our data showed that the expression of *SIRT1* did not differ between normal and malignant breast biopsies. This suggests that transcriptional changes for SIRT1 may not be implicated in breast cancer pathogenesis. This observation may be pertinent to the *p53* status of breast cancers, as *p53* is a substrate for *SIRT1* (Luo *et al*, 2001). Unlike the upregulation of telomerase, abnormalities in *p53* are seen in only 20–40% of breast cancers, suggesting that disruption of *p53* pathways may be of lesser importance in breast cancer pathogenesis (Mokbel *et al*, 2000; Borresen-Dale, 2003). These data do not, however, negate a role for *SIRT1* in breast cancer pathogenesis, as we looked at overall sirtuin transcriptional expression levels and not at any putative post-transcriptional regulation.

The lack of transcriptional change observed for *SIRT2* in this study may be a reflection of a cell cycle dependency in its expression (Dryden *et al*, 2003; North *et al*, 2003). It may be that more dynamic evaluation of changes in *SIRT2* expression at specific stages of the cell cycle may reveal abnormal patterns of *SIRT2* expression in breast cancer.

The clinical significance of the differences we have observed in sirtuin expression needs further investigation. Our study has indicated the potential utility of sirtuins as prognostic markers in breast cancer. The observation that *SIRT7* is increased in breast cancer tissue compared to normal breast tissue suggests that it may be related to breast cancer tumorigenesis. The discovery of a greater expression of *SIRT3* and *SIRT7*, within the prognostically poorer lymph node-positive breast cancers, suggests that overexpression of these sirtuins may be related to dissemination.

In summary, the heterogeneity of breast cancer behaviour is paralleled and may be explained by sirtuin expression. Specifically, molecular changes in *SIRT3* and *SIRT7* expression may contribute to tumour development and disease progression. This study shows that the study of sirtuins has a potential application in breast cancer diagnosis and prognosis, as well as in understanding disease biology.

## Figures and Tables

**Table 1:** The primer and probe sequences used in Taqman® PCR.

	**Forward primer (5′–3′)**	**Reverse primer (5′–3′)**	**Probe (5′ FAM–TAMRA 3′)**
*SIRT1*	TAGAGCCTCACATGCAAGCTCTA	GCCAATCATAAGATGTTGCTGAAC	ACTCCAAGGCCACGGATAGGTCCATATACTT
*SIRT2*	CCTCGCCTGCTCATCAACA	TCCTCCGAGGCCCATAATC	TGGCCAGTCGGACCCTTT
*SIRT3*	CATTCGGGCTGACGTGATG	AACCACATGCAGCAAGAACCT	TGCACCGGCGTTGTGAAGCC
*SIRT7*	CGTCCGGAACGCCAAATAC	GACGCTGCCGTGCTGATT	TGGTCGTCTACACAGGC
*p21*	GCAGACCAGCATGACAGATTTCTA	GCGGATTAGGGCTTCCTCTT	CACTCCAAACGCCGGCTGATCTTC
			
*p16*	CATAGATGCCGCGGAAGT	CCCGAGGTTTCTCAGAGCCT	CCTCAGACATCCCCGATTGAAAGAACC
			
*18S*	ACCTGGTTGATCCTGCCAGTAG	AGCCATTCGCAGTTTCACTGTAC	TCAAAGATTAAGCCATGCATGTCTAAGTACGCAC

**Table 2:** Summary of pathological prognostic factors of breast biopsies

					**ER status**		**Lvi**	**Grade**		
	**No**	**Age years (s.e.m.)**	**Nodal status**	**Size mm (s.e.m.)**	**+ve**	**−ve**		**II**	**III**	**NPI (s.e.m.)**
Normal	21	61.4 (3.1)	N/A	N/A	N/A	N/A		N/A		N/A
Cancer	24	62.3 (3.1)	+ve	30.7 (3.1)	9	3	6	4	8	4.1 (0.2)
			−ve	35.7 (3.1)	8	4	5	5	6	6.0 (0.3)

s.e.m.=standard error of the mean; N/A=not applicable; ER=oestrogen receptor; LVi=lymphovascular invasion; NPI=Nottingham Prognostic Index.
